# Effect of Mechanical Compression on Invasion Process of Malignant Melanoma Using *In Vitro* Three-Dimensional Cell Culture Device

**DOI:** 10.3390/mi10100666

**Published:** 2019-09-30

**Authors:** Takashi Morikura, Shogo Miyata

**Affiliations:** 1 Graduate School of Science and Technology, Keio University, 3-14-1 Hiyoshi, Yokohama 223-8522, Japan; dnngu-1elife@keio.jp; 2 Department of Mechanical Engineering, Faculty of Science and Technology, Keio University, 3-14-1 Hiyoshi, Yokohama 223-8522, Japan

**Keywords:** mechanical compression, malignant melanoma, cell invasion, *in vitro* cell culture model, melanin synthesis

## Abstract

Malignant melanoma in the plantar surface of the foot is subjected to various mechanical stimuli generated by daily human activity such as walking. Some studies have reported that mechanical compression affects the development and progression of melanoma. However, little is known about how mechanical compression affects the behavior of malignant melanoma cells in a physiological condition due to the complexity of the invasion mechanisms. In this study, we developed an *in vitro* three-dimensional cell culture device using microporous membrane in order to evaluate the effects of mechanical compression on the invasion process of malignant melanoma. Our results suggest that the invasion of melanoma cells under the compressive stress for 8 h of culture was promoted with the elongation of F-actin filaments compared to control groups, whereas there was no significant difference between both groups at 32 h of culture, with increasing cell death associated with promoting melanin synthesis. The results of this study contribute to the elucidation of the invasion mechanisms of malignant melanoma caused by mechanical stimulation.

## 1. Introduction

Malignant melanoma is the most aggressive type of cutaneous malignant tumor, and is one of the fastest increasing types of malignant tumor [1,2]. In recent years, the incidence of melanoma continues to increase in contrast to those of other tumors, and death attributed to melanoma is higher as compared to other solid tumors [3]. Compared to other malignant tumors that can be treated effectively even in advanced stages, malignant melanoma shows a poor response to chemotherapy, immunotherapy, and radiation therapy [4]. Current diagnostic methods for melanocytic lesions are limited, because they are based on histopathology and dermoscopy to evaluate wide morphological diversity and evolution patterns whose origin is still unknown [5,6]. In order to develop better therapeutic options, it is necessary to understand the physiological invasion mechanisms of malignant melanoma.

Malignant melanoma occurring in skin tissue is mainly associated with ultraviolet (UV) radiation exposure, but melanoma can also occur in specific areas of skin that are not exposed to UV light such as the palmoplantar surfaces. Recent research suggests that different pathogenic processes may be at work in the case of melanomas on the plantar surface [7,8]. Minagawa et al. reported that melanomas tended to develop in the areas of plantar surfaces subjected to larger mechanical stress, such as the rear and front regions of the plantar aspect [9]. Stucke et al. showed that mechanical stress, such as plantar pressure and shear stress, was higher in these two areas than in other areas of the plantar aspects [10]. Taken together, these findings suggest that mechanical stress could promote the formation of melanoma on the plantar aspect.

Some researchers recently reported that the micromechanical environment influenced the growth and physiological dynamics of tumor tissue [11,12,13,14,15]. Cancer cells are subjected to mechanical stress when they invade normal tissue. Previous studies reported that the mechanical stress not only directly regulated the proliferation and viability, but also induced genotypic and morphological changes of cancer cells [16,17,18,19,20,21,22,23]. The invasion of cancer cells has traditionally been studied with two-dimensional culture methods due to technical limitations. However, some researchers reported that invasive cells could differ in their morphology and mode of invasion depending on the dimensionality of the substrate [23,24,25,26]. Therefore, a two-dimensional culture model is not sufficient to simulate physiological conditions to study cancer cell invasion. Some researchers recently reported three-dimensional approaches using a multicellular cancer spheroid culture to study the invasion of cancer cells and the relationships between the mechanical environment and cell morphology [27,28,29,30].

However, a spheroid culture model is still not sufficient to simulate the mechanical environment and the invasion process of malignant melanoma. In the early period of malignant melanoma progression, melanoma cells commonly proliferate horizontally in the epidermis (radial growth phase). After the primary melanoma escapes the control of basal keratinocytes, they pass through the basement membrane with a phenotypic transition (vertical growth phase) [31,32]. Therefore, it is required to study the invasion mechanism in detail using a three-dimensional cell culture model that simulates the physiological three-dimensional condition of malignant melanoma.

Here, we established an *in vitro* three-dimensional cell culture model using a microporous membrane in order to evaluate the effects of mechanical compression on the invasion process of malignant melanoma. The main aim of this study is to elucidate how mechanical compression affects the behavior of malignant melanoma cells in a three-dimensional culture simulating physiological conditions.

## 2. Materials and Methods 

### 2.1. Cell Culture

The mouse malignant melanoma cell line B16F10 (RIKEN BioResource Center, Ibaraki, Japan) was used to establish an *in vitro* malignant melanoma model. B16F10 cells were thawed from a cryopreserved stock and subcultured twice in Dulbecco’s modified Eagle’s medium (DMEM) high glucose supplemented with 10% fetal bovine serum (FBS) and 1% antibiotics/antimycotics. The cells were maintained in a 5% CO_2_ atmosphere at 37 °C and passaged once in 2–3 days to avoid reaching confluence that causes cell-cell contact inhibition.

### 2.2. *In Vitro* Malignant Melanoma Model to Enable Imposing Static Compression and Monitoring of Cell Proliferation and Migration

**Figure 1:**
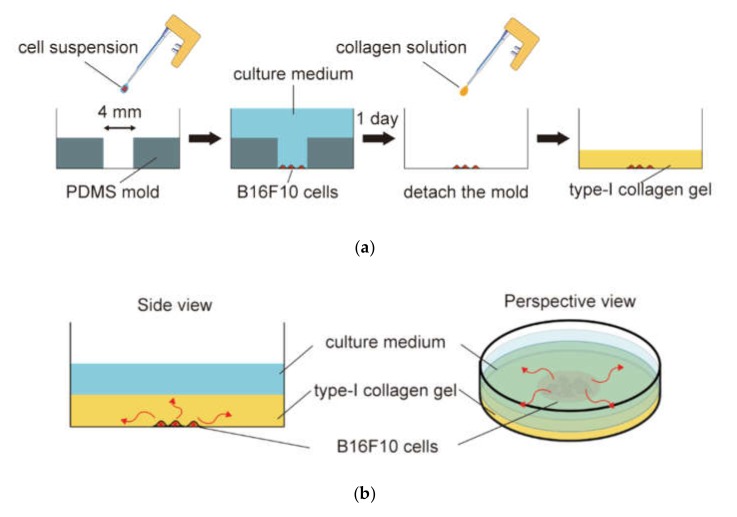
*In vitro* malignant melanoma model simulating the invasion process of tumor cells under three-dimensional skin-like conditions: (**a**) Fabrication of the three-dimensional malignant melanoma model. B16F10 cells were seeded in a 4-mm diameter cylindrical mold on a tissue culture dish. After one day of culture, the mold was removed from the dish and covered with neutralized type-I collagen gel to simulate skin tissue; (**b**) A schematic of the invasion process of B16F10 cells in the three-dimensional malignant melanoma model. The red arrows indicate the invasion of B16F10 cells into the collagen gel.

An *in vitro* malignant melanoma model was established by seeding the B16F10 cells under a type-I collagen gel layer. The B16F10 cells were seeded into 1.6 × 10^4^ cells/cm^2^ in a 4-mm diameter cylindrical mold on a tissue culture dish (Figure 1a) and cultured in DMEM high glucose with 10% FBS and 1% antibiotic/antimycotic at 37 °C in 5% CO_2_ for one day to confirm cell adhesion. The mold was removed from the dish after one day of culture. The adhered cells were washed with phosphate-buffered saline (PBS) and covered with neutralized type-I collagen gel to simulate skin tissue. Briefly, type-I collagen neutral solution was prepared to yield a final concentration of 2.4 mg/mL from acid soluble type-I collagen (I-AC, KOKEN, Tokyo, Japan). Then, the neutral type-I collagen solution was poured into the B16F10 cell-seeded dish to cover the cells with an approximately 1-mm thick collagen gel layer (Figure 1b). After polymerization at 37 °C for 20 min, an 8-μm pore size cell culture insert (BD Falcon Inc., East Rutherford, NJ, USA) was mounted on the gel layer.

**Figure 2:**
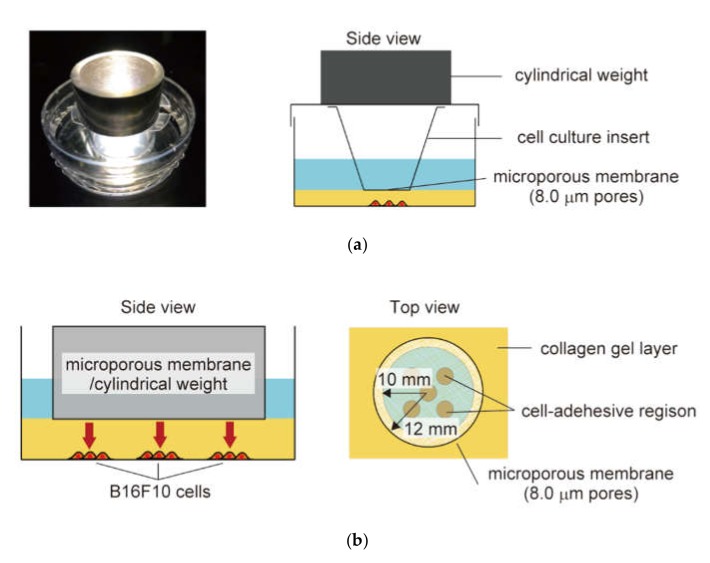
Schematic of the *in vitro* malignant melanoma model subjected to physiological compression: (**a**) The experimental set up for imposing static compression and monitoring cell proliferation and migration. A weight applies a constant compressive force to the B16F10 cells via the collagen gel layer and the microporous membrane of the cell culture insert; (**b**) The geometry of the *in vitro* malignant melanoma model. To assess cell proliferation and migration under constant compressive force, five cell-adhesive regions are located under the membrane of the cell culture insert. The behavior of B16F10 cells was monitored using phase-contrast and fluorescent microscopy.

The B16F10 cells were compressed for 32 h via the collagen gel layer using the microporous membrane-based cell culture insert with a ring-shaped weight (Figure 2). Oxygen and nutrients could diffuse toward the B16F10 cell-seeded area through the microporous membrane. To impose static compression on the gel-covered cells, a SUS304 stainless steel ring-shaped weight was put on the lid of the dish. According to the numerical analysis about the mechanical environment of malignant melanoma [15], a constant compressive stress of 7.7 × 10^2^ Pa was applied using the stainless steel weight. The behavior of cells in the malignant melanoma model could be observed through the ring-shaped weight throughout the culture period. A malignant melanoma model without weights was also prepared and maintained in a similar manner as a control group.

### 2.3. Quantification of Cell Invasion

The B16F10 cells in the malignant melanoma model were observed for 32 h using a phase-contrast microscope (CKX41, Olympus Inc., Tokyo, Japan) equipped with a CCD camera (DP73, Olympus Inc., Tokyo, Japan). Phase-contrast images were acquired at 0, 8, and 32 h after compression. Cell invasion was evaluated by the change in the occupied area of cultured cells in phase-contrast images. The cell-occupied area was measured by the ImageJ image processing and analysis software (NIH, MD, USA). The cell invasion area at each time $\left(CI_{t}\right)$ was calculated as follows:

(1) $$CI_{t}\;=A_{t}-A_{0}$$

where *A*_t_ stands for the cell-occupied area at each time, and *A*_0_ stands for the cell-occupied area at 0 h.

### 2.4. Evaluation of Cell Viability in Malignant Melanoma Model

To determine the effect of compression on cell viability in the malignant melanoma model, a fluorescence live/dead assay was performed. The cells were characterized with calcein-AM/propidium iodide (PI) double fluorescence staining. Cell viability was defined as the area of dead cells within the cell-occupied area measured by Image J.

### 2.5. Quantification of Melanin Synthesis

The amount and distribution of melanin in the malignant melanoma model were evaluated to determine the effect of compression on melanin synthesis in B16F10 cells. The melanin concentration was quantified based on the opacity of cells in phase-contrast images.

The opacity of cells in the phase-contrast image was evaluated using ImageJ software. First, the images were converted into 8-bit grayscale images. The parameter of melanin synthesis named as “melanin formation: *MF*” was defined as:

(2) $$MF=\sum_{i}mf_{i\;}$$

(3) $$mf_{i}\;=\;\frac{x_{i}-a_{0}}{a_{0}}\times 100\;(0<a_{0}<x_{i}\leq 255)$$

where $mf_{i\;}$ stands for the melanin formation at each pixel of the image, $x_{i\;}$ stands for the grayscale value at each pixel, $a_{0}$ stands for the grayscale value in the area without cells, and i stands for the location number of each pixel. The $\;a_{0}$ was calculated as an average of the grayscale value in four regions, which was extracted at random in the area without cells. The total amount of melanin in the model was evaluated using the *MF* value. The distribution of melanin was also evaluated by the $mf_{i\;}$ value of each pixel in the image.

### 2.6. Fluorescent Staining of F-Actin Cytoskeleton

To determine the effect of compression on the structure of the F-actin cytoskeleton at the outer edges of the cell-adhered area, the F-actin cytoskeleton was observed by fluorescence microscopy. Briefly, the cells in the malignant melanoma model were fixed with 4% paraformaldehyde for 10 min following permeabilization with 0.1% Triton X-100 in PBS for 5 min at room temperature. To stain F-actin filaments, the cells were incubated with 0.7% rhodamine-phalloidin (PHDR1, Cytoskeleton Inc, Denver, CO, USA) for 30 min at 37 °C. The F-actin filaments were visualized by an inverted microscope (CKX41, Olympus Inc. Tokyo, Japan) equipped with a CCD camera (DP73, Olympus Inc., Tokyo, Japan) and a fluorescent light source (U-LH50HG, Olympus Inc., Tokyo, Japan).

### 2.7. Statistical Analysis

Box and whisker plots were used to show quantitative data about cell invasion, viability, and melanin synthesis. These experimental data were examined for significant differences using Welch’s t-tests (cell invasion) and Student’s *t*-tests (cell viability and melanin synthesis). In all cases, *p* <0.05 was considered statistically significant.

## 3. Results

### 3.1. Effect of Static Compression on Invasion Process of Malignant Melanoma Cells

**Figure 3:**
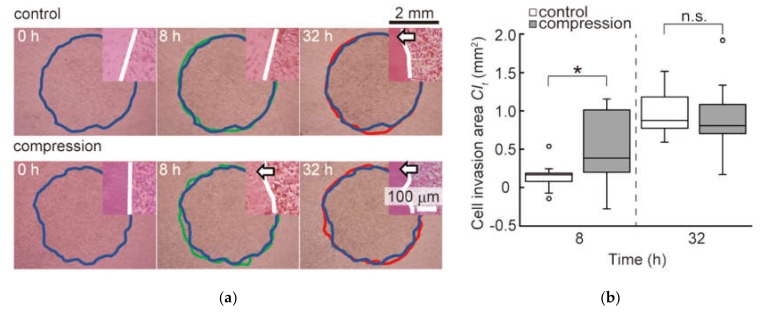
Invasion of B16F10 cells in the melanoma static compression model: (**a**) Microscopic images of B16F10 cells in control and compression groups. The migration of cells subjected to compressive stress was compared to control groups at each time point. The blue lines indicate the cell-adhered area at 0 h of culture, the green lines indicate one at 8 h of culture, and the red lines indicate one at 32 h of culture. The upper right images show the boundary region between the cell-adhered area and the collagen gel, with the white lines marking the boundary. The white arrows indicate the invasion of B16F10 cells into the collagen gel; (**b**) The cell invasion area $\;\left(CI_{t}\right)$ at each time (n = 13). Experimental data were examined for significant differences using Welch’s *t*-tests. * indicates a significant difference compared to control group (*p* < 0.05).

The microscopic images of B16F10 cells in control and compression groups are shown in Figure 3a. The cell population of both groups moved toward the outside of the cell-adhered area. The B16F10 cells in the compression groups migrated faster than those in control groups for 8 h after the start of compression. The cells in both groups invaded to reach similar distances from the center of the cell-attached region at 32 h. These results indicate that the compression promoted the invasion of cells in the malignant melanoma model. Figure 3b shows the change in the cell invasion area at each time $\left(CI_{t}\right)$ during the cultivation period. The cell invasion area in the compression groups at 8 h of culture increased significantly compared to the control groups. In other words, it was noted that the invasion of melanoma cells under the compressive stress for the first 8 h of culture was promoted in our melanoma model. In addition, there was no significant difference between both groups at 32 h of culture.

**Figure 4:**
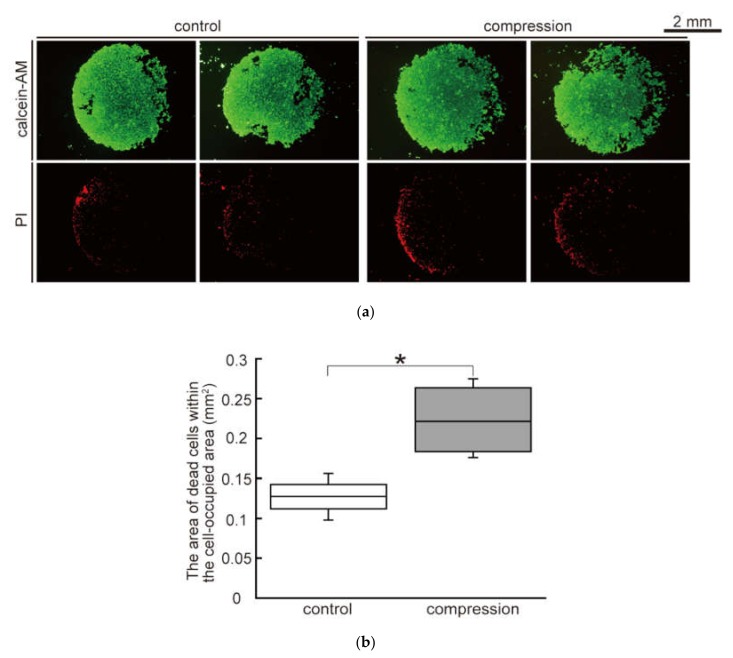
Effect of compression on cell viability in the malignant melanoma model: (**a**) Fluorescent microscopic images of B16F10 cells in the melanoma model stained by calcein-AM/PI (propidium iodide) at 32 h of culture. Live cells exposed to calcein-AM showed green fluorescence, while dead cells allowed PI to enter the cell membrane and label the cell nucleus with red fluorescence; (**b**) The area occupied by dead cells at 32 h of culture (n = 4). Experimental data were examined for significant differences using Student’s *t*-tests. * indicates a significant difference compared to control group (*p* < 0.05).

The fluorescent images of calcein-AM/PI staining of the control and compression groups at 32 h are shown in Figure 4a. Calcein-AM was used to distinguish live cells and PI marked dead cells. The results indicated that most cells in both groups were alive at 32 h of culture. The area occupied by dead cells at 32 h in both groups is shown in Figure 4b. It was found that the number of dead cells in the compression groups increased significantly compared to the ones in the control groups. 

**Figure 5:**
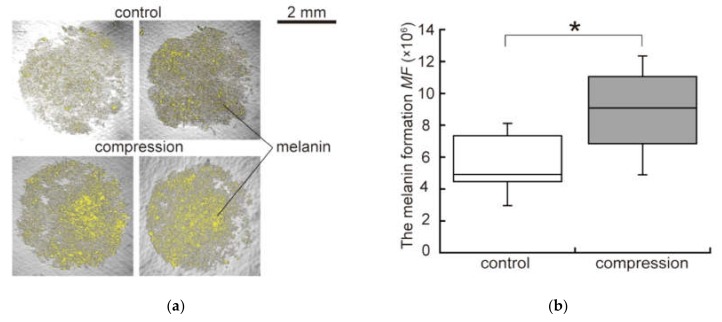
The difference of melanin synthesis in the malignant melanoma model: (**a**) Distribution of synthesized melanin in both groups at 32 h of culture. Yellow color in the images indicates the concentrated melanin; (**b**) The melanin formation (*MF*) at 32 h (n = 5). Experimental data were examined for significant differences using Student’s *t*-tests. * indicates a significant difference compared to control group (*p* < 0.05).

The representative images of melanin synthesis are shown in Figure 5a. The yellow color region in Figure 5a indicate the area where the $mf_{i\;}$ value is over zero and the melanin synthesis was promoted. It is notable that the melanin pigments were highly synthesized in the center region of the compression groups, whereas melanin pigment synthesis was scattered in control groups. Figure 5b shows the melanin formation (*MF)* at 32 h in both groups. This result indicates that the amount of melanin in the compression groups was significantly increased compared to that in the control groups.

### 3.2. Cytoskeletal Reorganization of Malignant Melanoma Cells and Elongation of F-actin Filaments

**Figure 6:**
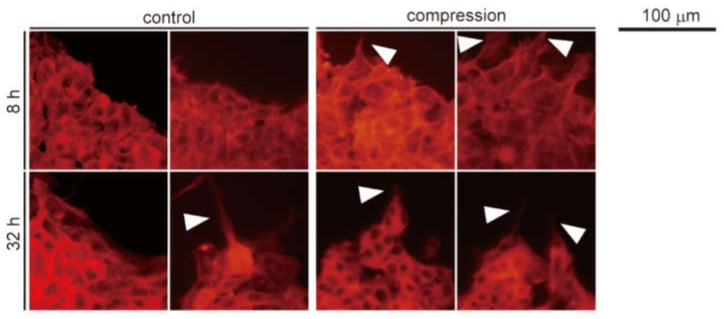
Fluorescent microscopic images of B16F10 cells in the melanoma model stained by rhodamine-phalloidin at each time point in the outer edges of the cell-adhered area. White arrowheads indicate an elongation of F-actin filaments.

The fluorescent images of rhodamine-phalloidin staining of control and compression groups at 8 h and 32 h after compression are shown in Figure 6. In the outer edges of the cell-adhered area, the B16F10 cells in the compression group at 8 h demonstrated elongated F-actin filaments, whereas those in the control groups did not. However, the elongated F-actin filaments were observed in both groups at 32 h of culture. As for the F-actin cytoskeleton of B16F10 cells in the outer edges of the cell-adhered area, the compression promoted cytoskeletal reorganization and induced the elongation of F-actin filaments in B16F10 cells. These findings indicate that mechanical compression affects cytoskeleton rearrangement in B16F10 cells. 

## 4. Discussion

Malignant melanoma is a very aggressive tumor that becomes chemoresistant and radiation-resistant following its transition to metastatic melanoma [33]. The incidence of malignant melanoma is still increasing worldwide, making it an urgent focus for research. It is commonly believed that melanocytic nevi may progress into melanoma, or alternatively dedifferentiate from melanocytes into melanoma [34]. It has been reported that melanomas with high grades of malignancy tend to occur on the plantar surface of the foot [9,10]. To address the current questions in the field, this study aimed to determine the effect of mechanical stress on the behavior of malignant melanoma cells. 

The results of our study revealed that compressive stimuli for 8 h promoted the invasion of melanoma cells, whereas there was no significant difference between compression and control groups at 32 h of the culture. As for cell proliferation, the doubling time of B16F10 cells is known to be 17.2 h [35]. Considering the doubling time of B16F10 cells, the expansion of the cell-adhered area at 8 h of culture was caused by cell migration. This result indicates that static compression for the first 8 h of culture promoted cell motility. Tse et al. reported that a constant stress of 7.7 × 10^2^ Pa, which is similar to the stress levels estimated in the microenvironment of the native tumor, did not significantly increase cell proliferation, but enhanced the motility of mammary breast tumor cells [21]. Our results at 8 h of culture are consistent with their results of the effect of mechanical stress on cell motility. Furthermore, our melanoma model with the use of different weights has possibilities to elucidate the relationship between compressive stress and cell response more quantitatively.

Due to the doubling time of B16F10 cells, the change in the cell-occupied area at 32 h was due to cell proliferation as well as migration [21]. According to the live/dead assay, the number of dead cells in the compression groups increased significantly compared to the control groups. This finding indicates that static compression for 32 h caused cell death. In general, mechanical stress directly regulates cell viability [17,23]. It was considered that static compression for 32 h might decrease cell viability and result in the lack of a significant difference regarding cell invasion between compression and control groups.

To elucidate the reason for cell death, we focused on the melanin pigments synthesized by B16F10 cells. The melanin synthesis in our malignant melanoma model was evaluated by the colorimetric analysis of microscopic images. From the results, the melanin synthesis of B16F10 cells in the compression groups was promoted compared to the one in the control groups. Melanin pigments are known as a factor that can promote cell death. Intermediate metabolites in melanin synthesis include highly reactive quinone compounds that promote the production of reactive oxygen species and oxidative DNA damage [36,37]. It was also reported that excess oxidative stress due to dysregulated melanin biosynthesis promoted cell death [38,39,40]. Taking into consideration all these findings, it was hypothesized that the increased melanin pigment synthesis evoked by the mechanical compression provided excess oxidative stress to the B16F10 cells and caused cell death at 32 h of culture. 

To evaluate changes in cell motility, the F-actin filaments of B16F10 cells in various areas of the melanoma model were also observed. According to our results, compressive stress for 8 h promoted cytoskeletal rearrangement and the elongation of F-actin filaments in cells in the outer area. It was reported that the elongation of F-actin filaments is related to enhanced cell motility [41]. Tse et al. reported that static compression caused an elongation of F-actin filaments in cancer cells to promote cell migration [21]. It is suggested that mechanical compression changes the morphology of B16F10 cells into the invasive phenotype; hence, static compression for 8 h promoted the invasion of cells toward the collagen gel layer.

## 5. Conclusions

The *in vitro* three-dimensional cell culture model of malignant melanoma was established to understand the effects of static compression on the invasion process of melanoma. From the results of this study, it is suggested that the invasion of melanoma cells under the compressive stress for 8 h of culture was promoted with the elongation of F-actin filaments compared to control groups. On the other hand, there was no significant difference between both groups at 32 h of culture, with increasing cell death associated with promoting melanin synthesis. Considering the results of this study, our melanoma model has the potential to elucidate the physiological relationship between the mechanical environment and the development of melanoma.
